# Influence of hormonal levels on atherosclerotic risk markers and platelet activation related to diabetic complications in patients with type 1 diabetes

**DOI:** 10.1007/s40618-026-02819-1

**Published:** 2026-01-31

**Authors:** Evangelia Baldimtsi, Bertil Ekman, Per A. Whiss, Jeanette Wahlberg

**Affiliations:** 1 Department of Activity and Health, and Department of Health, Medicine and Caring Sciences, Linköping University Hospital, Linköping University, Linköping, Sweden; 2 Department of Endocrinology in Linköping and Norrköping, and Department of Health, Medicine and Caring Sciences, Linköping University, Linköping, Sweden; 3 Department of Biomedical and Clinical Sciences, Division of Clinical Chemistry and Pharmacology, Linköping University, Linköping, Sweden; 4 Faculty of Medical Sciences, Örebro University, Örebro, Sweden

**Keywords:** Diabetes mellitus type 1, Insulin-like growth factor I, Matrix metalloproteinase 9, Tissue inhibitor of metalloproteinase-1, Platelet adhesiveness, Diabetic neuropathy, Adiponectin, Renin

## Abstract

**Background and Aims:**

Patients with childhood-onset type 1 diabetes (T1D) are at increased risk of developing microvascular complications, including neuropathy and nephropathy. Hormonal dysregulation and markers of atherosclerotic plaque instability and platelet activation may play key roles in the pathogenesis of these complications. The aim of this study was to investigate the impact of hormonal levels on atherosclerotic risk markers and platelet function, as well as to explore the association between diabetic neuropathy and nephropathy in individuals with childhood-onset type 1 diabetes.

**Methods:**

In this cross-sectional analysis of a longitudinal cohort, 34 individuals with childhood-onset type 1 diabetes (mean age 27.6 ± 4.2 years; diabetes duration 8.2 ± 5.6 years) were examined. S-IGF-I, long-term HbA1c, micro/macroalbuminuria, triiodothyronine and thyroxine, S-Cortisol, P-ACTH, P-Renin, sP-Selectin, P-MMP-9, P-TIMP-1, P-Adiponectin, and platelet adhesion to albumin, collagen, and fibrinogen were assessed. An abnormality in nerve conduction tests was defined as diabetic neuropathy.

**Results:**

S-IGF-I was negatively correlated with age (r = -0.36, *p* = 0.007), and with long-term HbA1c (r = -0.426, *p* = 0.019, corrected for age). IGF-I levels in patients diagnosed with clinical neuropathy (n = 6) were lower (123 ± 38 µg/L) than in patients without neuropathy (n = 26, 178 ± 56 µg/L, *p* = 0.029). S-IGF-I levels were also lower in patients with nephropathy (n = 7, 122 ± 28 µg/L) compared with patients without nephropathy (n = 27, 180 ± 60 µg/L, *p* = 0.02). S-IGF-I was negatively correlated with P-TIMP-1 (r = -0.44, p = 0.009), sP-Selectin (r = -0.53, p = 0.001), and positively correlated with platelet adhesion to fibrinogen (r = 0.38, *p* = 0.035). S-free-Triiodothyronine correlated negatively with P-MMP-9, (r = -0.46, *p* = 0.005), and P-MMP-/P-TIMP-1 ratio (r = -0.40, *p* = 0.018), and P-Adiponectin (r = -0.49, *p* = 0.018). P-Renin correlated negatively with P-Adiponectin (r = -0.34, *p* = 0.045).

**Conclusions:**

Low serum IGF-I levels were associated with the presence of diabetic neuropathy and nephropathy in young adults with type 1 diabetes. Additionally, both IGF-I and S-free-Triiodothyronine levels were linked to changes in platelet activation and atherosclerotic markers, suggesting that hormonal dysregulation may contribute to early vascular complications in this population.

## Introduction

Atherothrombotic mechanisms are key contributors to the development of complications in type 1 diabetes (T1D), leading to high rates of morbidity and an increased risk of premature mortality [1]. Although recent decades have seen significant reductions in all-cause, cardiovascular disease (CVD), and diabetes-related mortality across most age groups, mortality rates associated with diabetes in individuals aged 0–40 years have not shown the same improvement [2].

A critical factor influencing the progression and outcome of vascular complications in T1D is the stability of atherosclerotic plaques. This stability is regulated by various molecular mediators, including matrix metalloproteinases (MMPs), tissue inhibitors of MMPs (TIMPs) [3, 4], and adiponectin [5]. Dysregulated extracellular matrix (ECM) remodeling by MMPs has been implicated in the pathogenesis of vascular injury [6] and diabetic complications [7]. MMPs constitute a family of zinc- and calcium-dependent endopeptidases responsible for the degradation and remodeling of ECM components such as collagen, elastin, gelatin, and casein [7]. MMP-9, a member of the gelatinase subgroup, is of particular interest, which contains fibronectin type II-like domains that facilitate binding to gelatin, collagen, and laminin [8]. MMP-9 activity is tightly regulated by its endogenous inhibitor, TIMP-1 [8].

Elevated serum levels of MMPs, including MMP-9 and TIMP-1, have been documented in individuals with T1D [3], and these proteins are increasingly recognized as potential biomarkers of microvascular complications [9]. In parallel, selectins—cell adhesion molecules expressed by activated endothelial cells and platelets—play a pivotal role in leukocyte recruitment and inflammatory response initiation during vascular injury and hemostasis [10].

Hormonal dysregulation observed in T1D, which may influence both vascular remodeling and the development of complications, includes the growth hormone–insulin-like growth factor-I (GH–IGF-I) axis [11–13], thyroid function [14], adrenocortical activity [15], and the renin–angiotensin–aldosterone system (RAAS) [16].

Notably, serum IGF-I levels are frequently reduced in individuals with impaired endogenous insulin secretion [11, 12]. Similarly, reduced circulating IGF-I has been observed in patients with type 2 diabetes, with more pronounced reductions in older patients and those with longer disease duration [17, 18]. This early and persistent IGF-I deficiency in T1D may contribute to the increased severity of diabetic peripheral neuropathy (DPN) in this population relative to type 2 diabetes [17]. Evidence also suggests that low IGF-I levels are particularly associated with painful diabetic neuropathy, the most disabling form of DPN [19].

In addition to their role in ECM turnover, MMPs may influence cellular proliferation and repair mechanisms by modulating the bioavailability of IGFs [20], suggesting a complex interplay between hormonal factors and MMP activity in the progression of diabetic complications.

### Aim

The aim of this study was to investigate the relationships between circulating levels of IGF-I, thyroxine/triiodothyronine, ACTH, cortisol, renin, and various markers of atherosclerotic risk markers and platelet function in patients with type 1 diabetes, stratified by the presence of diabetic neuropathy and/or nephropathy.

## Methods

### Participants

Thirty-four patients with childhood onset of type 1 diabetes 27.6 ± 4.2 years of age (mean ± SD, range 20–35) and disease duration 18.2 ± 5.6 years (mean ± SD, range 10–30) were investigated. All patients were prescribed intensive insulin therapy by administration of insulin several times daily by either injection or a subcutaneous infusion pump, from the onset of diabetes. The cross-sectional study population consisted of participants originally deriving from a longitudinal cohort study [21, 22]. Pregnant patients were excluded from the study through active questioning during the inclusion process. Exclusion criteria included a history of neurological disease, metabolic diseases other than type 1 diabetes, alcohol or drug abuse, poor medication adherence, and the use of medications known to impair peripheral nerve function.

### Measurements

Medical records were retrieved, and all HbA1c values were extracted and converted to units according to the International Federation of Clinical Chemistry and Laboratory Medicine (IFCC, mmol/mol) and to the National Glycohemoglobin Standardization Program (NGSP, %). Long-term metabolic control at both baseline and follow-up was estimated using the mean weighted HbA1c (wHbA1c). Specifically, all available HbA1c values from the time of diabetes diagnosis up to the time of the baseline and follow-up examination were included. To account for the irregular intervals between measurements, mean wHbA1c was calculated as the area under the curve (AUC) of time versus HbA1c, divided by the total follow-up time for each individual. Long-term metabolic control,is referred to as HbA1c from this point onwards. S-free-Thyroxine, S-free-Triiodothyronine, P-ACTH, S-Cortisol, S-IGF-I, and P-Renin, and B-HbA1c were analyzed at the Clinical Chemistry Laboratory at the University Hospital of Linköping, Sweden.

Alongside routine biochemical analyses conducted at the Clinical Chemistry Laboratory on the day of blood collection, samples were centrifuged at 1500 × g for 15 min and plasma was aliquoted and stored at − 80 °C at the Linköping Biobank Facility, University Hospital, Sweden. The following markers were thereafter analysed in thawed plasma at the Division of Clinical Chemistry and Pharmacology, Linköping University, Sweden, as various components and functions of the atherosclerotic process: MMP-9 (collagen breakdown), its tissue inhibitor TIMP-1(inhibition of MMP), Adiponectin (plaque stabilization), and soluble P-selectin (platelet activation) (sP-selectin) were analyzed in plasma using enzyme immunoassays provided by R&D Systems Europe Ltd (Abingdon, England).

IGF-I was measured by a solid-phase, enzyme-labeled chemiluminescent immunometric assay (IMMULITE® 2000 immunoassay system, Siemens Healthcare Diagnostics, Mölndal, Sweden).

Static platelet adhesion to albumin, collagen, and fibrinogen was studied in 96-well microplates using a method previously described [23]. Briefly, venous blood was collected in sodium heparin tubes (BD Vacutainer®). Platelet-rich plasma (PRP) was prepared, and the proportion of platelet adhesion was investigated by incubating PRP in protein-coated microplate wells for one hour at room temperature. After incubation, the microplates were washed, followed by the detection of percent adhered platelets by an enzymatic/spectrophotometric procedure.

Both routine biomarkers and atherosclerotic risk markers and platelet activation were analyzed cross-sectionally from the last follow up in this longitudinal study.

An electrodiagnostic evaluation was used for diagnosing diabetic peripheral neuropathy (DPN). This involved using surface electrodes to measure nerve conduction velocities (NCV) in sensory and motor nerves. The specific nerves tested included the sural nerve for sensory nerve conduction velocity (SCV) and sensory nerve action potential (SNAP) and the peroneal nerve for motor nerve conduction velocity (MCV) and compound muscle action potential amplitude (CMAP). The neuropathy symptom assessment score (NSA) was used to screen for symptoms like paraesthesia, numbness, allodynia, and pain in the lower extremities. In addition, the neuropathy impairment assessment (NIA) evaluated clinical signs of neuropathy through sensory screenings (temperature, pinprick, vibration) and reflex tests.

Subclinical neuropathy was identified solely by abnormal nerve conduction tests without any symptoms or signs meanwhile clinical DPN was diagnosed when both abnormal nerve conduction tests and symptoms or signs of neuropathy were present. Painful diabetic neuropathy was characterized by spontaneous pain, mechanical hyperalgesia, and tactile allodynia, and defined by abnormalities in nerve conduction tests in at least two separate nerves along with an NSA score of 1 or higher[24]. Diabetic nephropathy was characterized by increased urinary albumin excretion in the absence of other renal diseases and was categorized into stages: microalbuminuria was defined as an albumin excretion rate of 20–200 mg/min or an albumin/ creatinine ratio of 3–30 mg/mmol. Macroalbuminuria was defined as an albumin excretion rate > 200 mg/min or albumin/creatinine ratio > 30 mg/mmol.

### Statistical analysis

Mean and standard deviation (SD) and median 25th and 75th percentiles are reported for continuous variables, while numbers and percentages are reported for categorical variables. Comparisons between groups of data were done using unpaired Student´s t-test and within groups with paired Student´s t-test. For the estimation of linear associations, the Pearson correlation coefficient was calculated. In the case of non-normal data, the Mann–Whitney U test was used, and within groups, comparisons were made with the Wilcoxon rank sum test. P-values less than 0.05 were considered statistically significant.

Statistics were calculated on a PC using SPSS version 29.0 (IBM Corp, Armonk, NY, USA).

## Results

### Demographics

Thirty-four patients (53% male) with childhood-onset type 1 diabetes were investigated (mean age 27.6 ± 4.2 years, range 20–35 years; mean disease duration 18.2 ± 5.6 years, range 10–30 years) (Table 1). In addition to prescribed intensive insulin therapy, two patients were receiving ACE inhibitors, two were on statins, and one was on levothyroxine at the time of evaluation. In this cross-sectional study, patients were divided into two groups based on their smoking habits: 10 active smokers and 24 non-smokers (including 11 past smokers and 13 who had never smoked). No significant differences were observed between the two groups in terms of MMP-9 levels or other atherosclerotic risk markers.

### Clinical neuropathy and nephropathy

In all, eight patients were diagnosed with DPN, and of those, six had an NSA ≥ 1 and a high NIA score, indicating clinical neuropathy. On average, NIA was 23 points for the patients with clinical DPN and 11 points for the patients without DPN or subclinical neuropathy.The mean age in the group of patients with clinical DPN was 6 years higher compared to the group without DPN. Among the symptoms of neuropathy, paraesthesia and allodynia were most common, reported by five patients, while one patient had symptoms of pain. Patients with clinical DPN had longer diabetes duration than patients without clinical DPN *p* = 0.016. Nerve conduction variables measured as standard deviation score (SDS) were lower in peroneal MCV, sural SCV, and SNAP in patients with clinical neuropathy.

Seven patients were diagnosed with microalbuminuria, but none in our cohort had macroalbuminuria.

### S-IGF-I and platelet and atherosclerotic markers

**Fig. 1:**
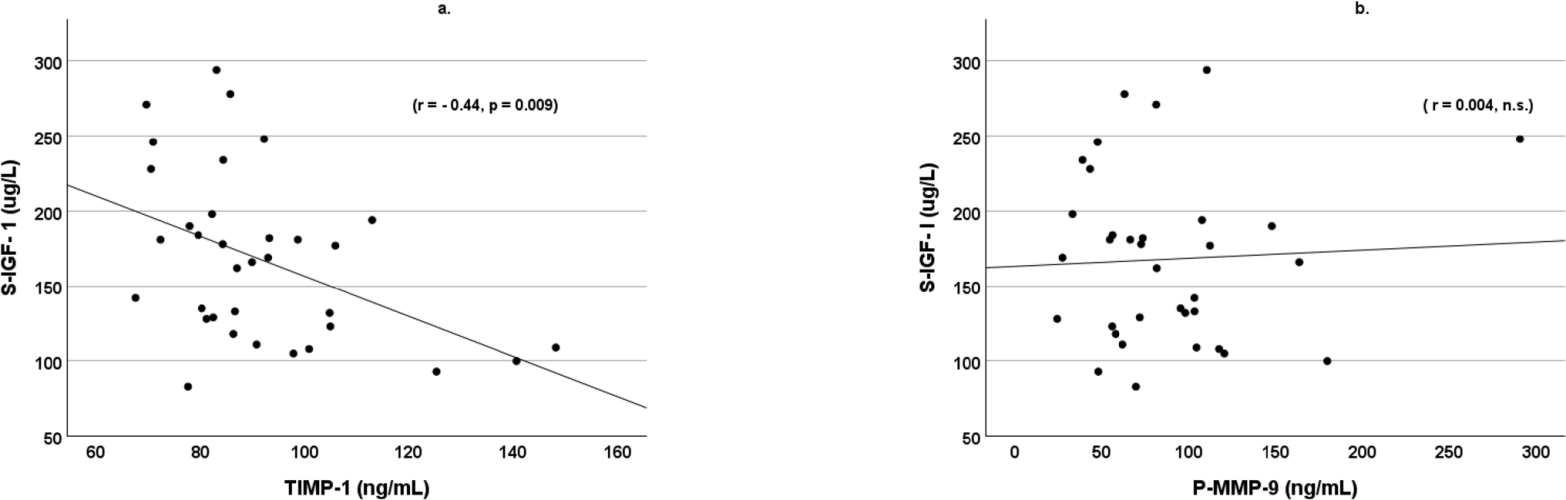
S-IGF-I correlated negatively with P-TIMP-1, r = -0.44, p = 0.009 (**a**), while no correlation was found with P-MMP-9 (**b**). Patients with type 1 diabetes (n = 34)

**Fig. 2:**
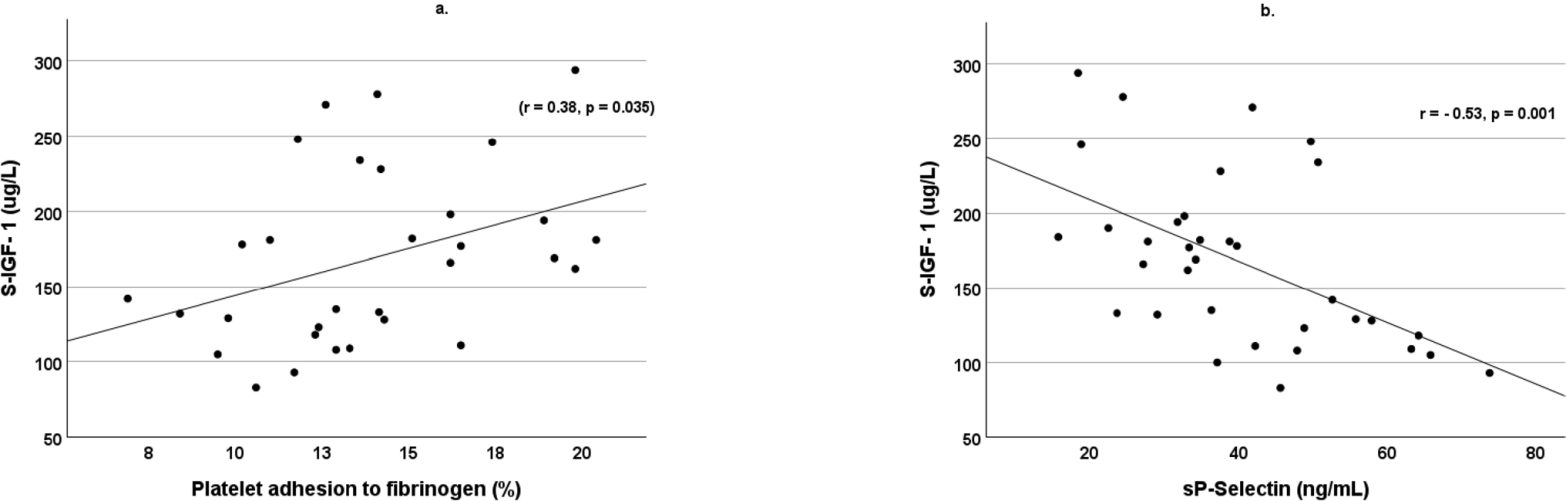
S-IGF-I correlated positively with platelet adhesion to fibrinogen, r = 0.38, p = 0.035 (**a**), and negatively to sP-Selectin r = -0.53, p = 0.001 (**b**). Patients with type 1 diabetes (n = 34)

**Fig. 3:**
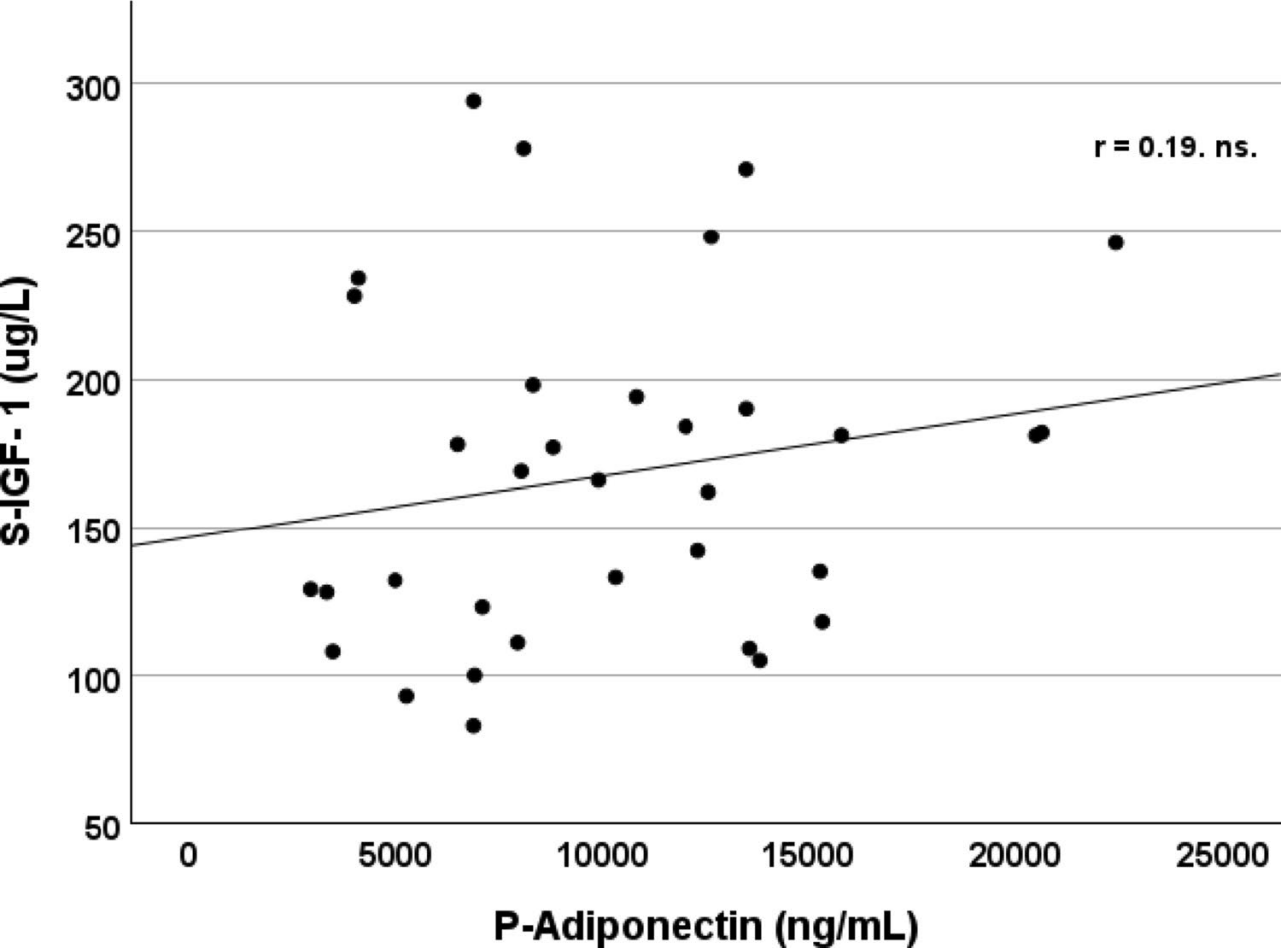
No relationship between S-IGF-I and P-Adiponectin was found. Patients with type 1 diabetes (n = 34)

S-IGF-I was negatively correlated with P-TIMP-1, (r = -0.44, *p* = 0.009, Fig. 1a), while no correlation was found with P-MMP-9 (Fig. 1b) or P-MMP-9/P-TIMP-1 ratio (r = 0.188, *p* = 0.287). S-IGF-I was positively correlated with platelet adhesion to fibrinogen, (r = 0.38, *p* = 0.035, Fig. 2a), and negatively to sP-Selectin (r = -0.53, *p* = 0.001, Fig. 2b). No relationship between S-IGF-I and P-Adiponectin was found (Fig. 3). Platelet adhesion to albumin on collagen surface showed no relationship to the S-IGF-I level (Data not shown).

### S-IGF-I and diabetic complications

S-IGF-I was negatively correlated with age (r = -0.36, *p* = 0.007), and negatively correlated with long-term HbA1c corrected for age, (r = -0.426, *p* = 0.019). IGF-I levels in patients diagnosed with clinical neuropathy (n = 6) were lower, 123 ± 38 µg/L, than in patients without neuropathy, (178 ± 56 µg/L, *p* = 0.029). S-IGF-I levels were also lower in patients with nephropathy (n = 7), 124 ± 25 µg/L, compared with patients without nephropathy, (180 ± 60 µg/L, *p* = 0.02).

### S-free-thyroxine, S-free-triiodothyronine, S-cortisol and P-renin and the atherosclerotic markers

No significant correlations were found between S-free-Thyroxine and the atherosclerotic markers, while S-free-Triiodothyronine correlated negatively with P-MMP-9 (r = -0.46, *p* = 0.005), and P-MMP-9/P-TIMP-1 ratio (r = -0.40, *p* = 0.018), and P-Adiponectin (r = -0.49, *p* = 0.018). P-Renin correlated negatively with P-Adiponectin, (r = -0.34, *p* = 0.045). No significant correlations were found for P-Cortisol or P-ACTH and the atherosclerotic markers.

## Discussion

Our findings support the concept that type 1 diabetes is characterized by alterations in several basal hormone systems, which may contribute to the development of diabetic complications through effects on atherosclerotic factors and platelet function. In particular, low circulating insulin-like growth factor I (IGF-I) was associated with diabetic complications, consistent with previous long-term follow-up studies [13]. Moreover, IGF-I was associated with alterations in sP-selectin and platelet adhesion, as well as TIMP-1. Metalloproteinases play an important role in the maintenance of balance between collagen biosynthesis and degradation in tissues [6]. Lower IGF-I together with increased sP-selectin and TIMP-1 may be connected to increased plaque activity, whereas the positive correlation of IGF-1 with platelet adhesion to fibrinogen may indicate that IGF-I may support the function of platelets to constitute vascular integrity in blood vessels.

Hormonal alterations affecting thyroid function, adrenocortical activity, the GH–IGF-I axis, and the renin–angiotensin–aldosterone system (RAAS) may all contribute to the development of microvascular complications in T1D [15, 16, 25, 26]. Atherosclerosis, a central contributor to macrovascular complications, is characterized by lipid accumulation, inflammation, and smooth muscle cell infiltration into the vessel wall, leading to plaque formation and, potentially, plaque rupture [27]. The stability of these atherosclerotic plaques is influenced by various enzymes, notably matrix metalloproteinases (MMPs), which degrade extracellular matrix components [3, 4]. MMPs are regulated by tissue inhibitors of metalloproteinases (TIMPs), with TIMP-1 reflecting MMP-9 activity [4]. High glucose levels cause non-enzymatic glycation of proteins, forming advances glycation end products (AGE), which in turn stimulate the expression and activity of MMPs and TIMPs[28]. Several ligands of the receptor for AGE (RAGE) are elevated in diabetic vasculature. For instance, in diabetic mice, MMP-9 expression and activity are increased by up to fivefold in the aorta [29]. Several studies have also reported elevated MMP-9 levels in patients with T1D, both with and without retinopathy [3]. Furthermore, an increased MMP-9/TIMP-1 ratio has been implicated in plaque instability [7, 8]. In a recent prospective study, elevated TIMP-1 levels were associated with neuropathy and macroalbuminuria, while increased MMP-9 was observed in patients with microalbuminuria [30].

Adiponectin, secreted by adipocytes, appears to exert a stabilizing effect on atherosclerotic plaques [5]. In the current study, adiponectin levels were inversely associated with IGF-I, plasma renin, and free-triiodothyronine levels, suggesting that long-standing T1D, characterized by intra-portal insulin deficiency, is linked with broad hormonal dysregulation.

Regarding platelet function, P-selectin—a cell adhesion molecule expressed on activated endothelial cells and platelets—was found in its soluble form (sP-selectin) to be negatively correlated with IGF-I levels. Prior studies have shown elevated sP-selectin levels in T1D patients with neuropathy [30]. Conversely, platelet adhesion to fibrinogen was positively associated with IGF-I, consistent with previous findings that IGF-I enhances platelet aggregation in both healthy controls and individuals with type 2 diabetes [31]. This effect was more pronounced in diabetic patients and correlated positively with HbA1c levels, supporting the notion that IGF-I influences platelet activation and adhesion, likely through fibrinogen bridging mechanisms.

Thyroid disorders affect different vascular territories. Hypothyroidism appears to contribute primarily to atherosclerosis and arterial thrombosis, while hyperthyroidism predisposes to venous thromboembolism. Thyroid hormones have a complex role in platelet regulation since physiological levels of thyroxine have been reported to activate platelets, whereas triiodothyronine appears to not activate platelets[32].In the present study, free-triiodothyronine correlated negatively with MMP-9, MMP-9/TIMP-1 ratio and adiponectin indicating that triiodothyronine is not involved in driving the atherosclerotic or platelet activating process, but rather the opposite. This is consistent with reports indicating that coagulation factors are primarily involved in thrombosis associated with hyperthyroidism [33].

IGF-I plays crucial roles in cell proliferation, differentiation, survival, and collagen biosynthesis [34]. Its activity and bioavailability are modulated by IGF-binding proteins (IGFBPs) [35]. In T1D, reduced portal insulin levels result in hepatic growth hormone resistance and subsequently decreased IGF-I production [11]. Peripheral insulin administration does not fully restore IGF-I levels, likely due to the absence of portal insulin delivery [36]. Studies indicate that intra-portal insulin infusion or IGF-I replacement therapy can partially correct these deficiencies [36, 37].

The RAAS is central to the pathophysiology of diabetic kidney disease. Hyperglycemia activates local RAAS pathways, affecting podocyte structure and basement membrane integrity. RAAS inhibition remains one of the most effective strategies for delaying renal disease progression in diabetes. Of particular interest, in vitro studies have shown that angiotensin receptor blockers, such as Olmesartan, can attenuate MMP-9 expression and activity induced by advanced glycation end products [38], further implicating RAAS in extracellular matrix remodeling and nephropathy.

A major strength of the present study is its extended 20-year follow-up, including serial nerve conduction assessments and hormonal evaluations. Longitudinal HbA1c data from the time of T1D diagnosis further enriches the dataset. However, the relatively small sample size and potential selection bias, where patients with suspected neuropathy may have been more inclined to participate, are limitations. Moreover, while significant associations were identified, causality cannot be definitively established due to multiple comparisons and other complications not studied like enteropathy [39]. Future prospective studies with larger cohorts are necessary to elucidate further the complex interplay of hormonal regulation and diabetic complications.

Conclusions: Low IGF-I levels were associated with diabetic complications. Free triiodothyronine and IGF-I levels were associated with altered platelet activation and an imbalance in plaque stability markers in young adults with type 1 diabetes. Further investigations are necessary to determine whether normalization of these hormones provides benefits to patients with type 1 diabetes.

## Data Availability

The data generated or analyzed during this study are available from the corresponding author upon reasonable request.

## Declarations

### Approval of the research protocol

The research protocol was approved by the Research Ethics Committee, Linköping, Sweden, with number Dnr M053-07.

## References

[1] Snell-Bergeon JK, Nadeau K (2012) Cardiovascular disease risk in young people with type 1 diabetes. J Cardiovasc Transl Res 5(4):446–462 22528676 10.1007/s12265-012-9363-x

[2] Harding JL et al (2016) Age-specific trends from 2000-2011 in all-cause and cause-specific mortality in type 1 and type 2 diabetes: a cohort study of more than one million people. Diabetes Care 39(6):1018–1026 27208325 10.2337/dc15-2308

[3] Jacqueminet S et al (2006) Elevated circulating levels of matrix metalloproteinase-9 in type 1 diabetic patients with and without retinopathy. Clin Chim Acta 367(1–2):103–107 16426593 10.1016/j.cca.2005.11.029

[4] Visse R, Nagase H (2003) Matrix metalloproteinases and tissue inhibitors of metalloproteinases: structure, function, and biochemistry. Circ Res 92(8):827–839 12730128 10.1161/01.RES.0000070112.80711.3D

[5] Pereira RI et al (2012) Adiponectin dysregulation and insulin resistance in type 1 diabetes. J Clin Endocrinol Metab 97(4):E642–E647 22278421 10.1210/jc.2011-2542 PMC3319187

[6] Galis ZS, Khatri JJ (2002) Matrix metalloproteinases in vascular remodeling and atherogenesis: the good, the bad, and the ugly. Circ Res 90(3):251–262 11861412

[7] Kadoglou NP et al (2005) Matrix metalloproteinases and diabetic vascular complications. Angiology 56(2):173–189 15793607 10.1177/000331970505600208

[8] Peeters SA et al (2015) Plasma levels of matrix metalloproteinase-2, -3, -10, and tissue inhibitor of metalloproteinase-1 are associated with vascular complications in patients with type 1 diabetes: the EURODIAB prospective complications study. Cardiovasc Diabetol 14:31 25848912 10.1186/s12933-015-0195-2 PMC4355971

[9] Papadopoulou-Marketou N et al (2021) Plasma levels of tissue inhibitor of metalloproteinase-1 in patients with type 1 diabetes mellitus associate with early diabetic neuropathy and nephropathy. Diab Vasc Dis Res 18(2):14791641211002470 33775157 10.1177/14791641211002470 PMC8481743

[10] McEver RP (2015) Selectins: initiators of leucocyte adhesion and signalling at the vascular wall. Cardiovasc Res 107(3):331–339 25994174 10.1093/cvr/cvv154 PMC4592324

[11] Ekman B, Nystrom F, Arnqvist HJ (2000) Circulating IGF-I concentrations are low and not correlated to glycaemic control in adults with type 1 diabetes. Eur J Endocrinol 143(4):505–510 11022197 10.1530/eje.0.1430505

[12] Hedman CA et al (2004) Residual beta-cell function more than glycemic control determines abnormalities of the insulin-like growth factor system in type 1 diabetes. J Clin Endocrinol Metab 89(12):6305–6309 15579794 10.1210/jc.2004-0572

[13] Palta M et al (2014) The trajectory of IGF-1 across age and duration of type 1 diabetes. Diabetes Metab Res Rev 30(8):777–783 24845759 10.1002/dmrr.2554 PMC4236234

[14] Gauthier BR et al (2020) Thyroid hormones in diabetes, cancer, and aging. Aging Cell 19(11):e13260 33048427 10.1111/acel.13260 PMC7681062

[15] Simunkova K et al (2010) Adrenocortical function in young adults with diabetes mellitus type 1. J Steroid Biochem Mol Biol 122(1–3):35–41 20433924 10.1016/j.jsbmb.2010.04.017

[16] Ambinathan JPN et al (2021) Relationships between inflammation, hemodynamic function and RAAS in longstanding type 1 diabetes and diabetic kidney disease. J Diabetes Complications 35(5):107880 33678512 10.1016/j.jdiacomp.2021.107880

[17] Ishii DN (1995) Implication of insulin-like growth factors in the pathogenesis of diabetic neuropathy. Brain Res Brain Res Rev 20(1):47–67 7711767 10.1016/0165-0173(94)00005-a

[18] Migdalis IN et al (1995) Insulin-like growth factor-I and IGF-I receptors in diabetic patients with neuropathy. Diabet Med 12(9):823–827 8542744 10.1111/j.1464-5491.1995.tb02086.x

[19] Morgado C et al (2011) Changes in serotoninergic and noradrenergic descending pain pathways during painful diabetic neuropathy: the preventive action of IGF1. Neurobiol Dis 43(1):275–284 21515376 10.1016/j.nbd.2011.04.001

[20] Fowlkes JL et al (2004) Regulation of insulin-like growth factor (IGF)-I action by matrix metalloproteinase-3 involves selective disruption of IGF-I/IGF-binding protein-3 complexes. Endocrinology 145(2):620–626 14605000 10.1210/en.2003-0636

[21] Hyllienmark L et al (2013) Early electrophysiological abnormalities and clinical neuropathy: a prospective study in patients with type 1 diabetes. Diabetes Care 36(10):3187–3194 23723354 10.2337/dc12-2226 PMC3781488

[22] Hyllienmark L et al (2001) Nerve conduction defects are retarded by tight metabolic control in type I diabetes. Muscle Nerve 24(2):240–246 11180207 10.1002/1097-4598(200102)24:2<240::aid-mus90>3.0.co;2-2

[23] Eriksson AC, Whiss PA (2005) Measurement of adhesion of human platelets in plasma to protein surfaces in microplates. J Pharmacol Toxicol Methods 52(3):356–365 16005248 10.1016/j.vascn.2005.06.002

[24] Tesfaye S et al (2010) Diabetic neuropathies: update on definitions, diagnostic criteria, estimation of severity, and treatments. Diabetes Care 33(10):2285–2293 20876709 10.2337/dc10-1303 PMC2945176

[25] Kim MS, Lee DY (2015) Insulin-like growth factor (IGF)-I and IGF binding proteins axis in diabetes mellitus. Annals Pediatric Endocrinol Metabolism 20(2):69–73 10.6065/apem.2015.20.2.69 PMC4504992 26191509

[26] Wysocka-Mincewicz M et al (2021) Thyroid hormones, peripheral white blood count, and dose of basal insulin are associated with changes in nerve conduction studies in adolescents with type 1 diabetes. Metabolites. 10.3390/metabo11110795 34822453 10.3390/metabo11110795 PMC8619894

[27] Libby P et al (2019) Atherosclerosis Nat Rev Dis Primers 5(1):56 31420554 10.1038/s41572-019-0106-z

[28] Liu J, Pan S, Wang X et al (2023) Role of advanced glycation end products in diabetic vascular injury: molecular mechanisms and therapeutic perspectives. Eur J Med Res 28:553 38042909 10.1186/s40001-023-01431-w PMC10693038

[29] Bucciarelli LG, Wendt T, Qu W, Lu Y, Lalla E, Rong LL, Goova MT, Moser B, Kislinger T, Lee DC, Kashyap Y, Stern DM, Schmidt AM (2002) RAGE blockade stabilizes established atherosclerosis in diabetic apolipoprotein E-null mice. Circulation 106(22):2827–2835. 10.1161/01.cir.0000039325.03698.36. (**PMID: 12451010**) 12451010 10.1161/01.cir.0000039325.03698.36

[30] Baldimtsi E, Whiss PA, Wahlberg J (2023) Systemic biomarkers of microvascular alterations in type 1 diabetes associated neuropathy and nephropathy - a prospective long-term follow-up study. J Diabetes Complications 37(12):108635 37989066 10.1016/j.jdiacomp.2023.108635

[31] Gligorijevic N, Robajac D, Nedic O (2019) Enhanced platelet sensitivity to IGF-1 in patients with type 2 diabetes mellitus. Biochemistry (Mosc) 84(10):1213–1219 31694517 10.1134/S0006297919100109

[32] Davis PJ, Mousa SA, Schechter GP (2018) New interfaces of thyroid hormone actions with blood coagulation and thrombosis. Clin Appl Thromb Hemost 24(7):1014–1019. 10.1177/1076029618774150 29742907 10.1177/1076029618774150 PMC6714741

[33] Elbers LPB, Fliers E, Cannegieter SC (2018) The influence of thyroid function on the coagulation system and its clinical consequences. J Thromb Haemost 16(4):634–645. 10.1111/jth.13970 29573126 10.1111/jth.13970

[34] Hakuno F, Takahashi SI (2018) IGF1 receptor signaling pathways. J Mol Endocrinol 61(1):T69–T86 29535161 10.1530/JME-17-0311

[35] Baxter RC, Martin JL (1989) Binding proteins for the insulin-like growth factors: structure, regulation and function. Prog Growth Factor Res 1(1):49–68 2485012 10.1016/0955-2235(89)90041-0

[36] Hedman CA et al (2014) Intraperitoneal insulin delivery to patients with type 1 diabetes results in higher serum IGF-I bioactivity than continuous subcutaneous insulin infusion. Clin Endocrinol (Oxf) 81(1):58–62 23865977 10.1111/cen.12296

[37] Conti E et al (2011) IGF-1 and atherothrombosis: relevance to pathophysiology and therapy. Clin Sci Lond 120(9):377–402 21244364 10.1042/CS20100400

[38] Kamioka M et al (2010) Blockade of renin-angiotensin system attenuates advanced glycation end products-mediated signaling pathways. J Atheroscler Thromb 17(6):590–600 20379053 10.5551/jat.3624

[39] D’Addio F, La Rosa S, Maestroni A, Jung P, Orsenigo E, Ben Nasr M, Tezza S, Bassi R, Finzi G, Marando A, Vergani A, Frego R, Albarello L, Andolfo A, Manuguerra R, Viale E, Staudacher C, Corradi D, Batlle E, Breault D, Secchi A, Folli F, Fiorina P (2015) Circulating IGF-I and IGFBP3 levels control human colonic stem cell function and are disrupted in diabetic enteropathy. Cell Stem Cell 17(4):486–498. 10.1016/j.stem.2015.07.010 26431183 10.1016/j.stem.2015.07.010 PMC4826279

